# Hypomethylating agents plus venetoclax in younger acute myeloid leukemia: Meta‐analysis of a shifting treatment paradigm

**DOI:** 10.1002/cncr.70372

**Published:** 2026-03-31

**Authors:** Salvatore Perrone, Laura De Fazio, Sebastian Monachetti, Matteo Molica

**Affiliations:** ^1^ Department of Hematology S. M. Goretti Hospital Polo Universitario Pontino Latina Italy; ^2^ Department of Hematology‐Oncology Azienda Universitaria Ospedaliera Renato Dulbecco Catanzaro Italy; ^3^ Department of Translational and Precision Medicine Sapienza University Rome Italy

**Keywords:** AML, azacitidine, decitabine, meta‐analysis, venetoclax

## Abstract

**Background:**

In younger, fit patients with acute myeloid leukemia (AML), intensive chemotherapy (IC) followed by consolidation or allogeneic hematopoietic stem cell transplantation (HSCT) is the standard approach. The authors performed a systematic review and meta‐analysis to evaluate younger patients with AML treated with hypomethylating agents (HMA) plus venetoclax.

**Methods:**

This systematic review and meta‐analysis was conducted in accordance with Preferred Reporting Items for Systematic Reviews and Meta‐Analyses guidelines. MEDLINE and the Cochrane Library were systematically searched through February 2026. Studies included AML patients with a median age <70 years treated with HMA/venetoclax. Primary outcomes were complete remission (CR/CRi) rate, measurable residual disease (MRD) negativity, 1‐year overall‐survival (OS), 1‐year event‐free survival (EFS), and rates of HSCT.

**Results:**

Eight studies (two randomized controlled trials, two phase 2 trials, and four real‐world studies), comprising 429 patients with a mean age of 54 years, were included. The pooled CR/CRi rate was 66% (95% confidence interval [CI], 48%–85%), with an MRD‐negative rate of 69% (95% CI, 49%–90%). The pooled 1‐year OS was 75% (95% CI, 63%–86%), exceeding Surveillance, Epidemiology, and End Results database cohorts (62%). The 1‐year EFS was 59% (95% CI, 53%–65%), with low between‐study heterogeneity. Overall, 66% of patients successfully proceeded to HSCT. Meta‐regression analyses suggested a trend toward improved EFS and OS in studies using decitabine than azacitidine.

**Conclusions:**

In younger patients with AML, HMA plus venetoclax yielded high response rates, MRD negativity, and a substantial proportion of patients proceeding to HSCT. These findings support HMA/venetoclax as an effective induction strategy in selected younger patients and provide a rationale for prospective randomized trials comparing this approach with IC‐based regimens.

## Abstract 2

In a meta‐analysis of eight studies, 429 young acute myeloid leukemia (AML) patients (mean age of 54 years) treated with hypomethylating agents (HMA)/venetoclax the pooled complete remission rate was 66% with a 1‐year overall survival was 75%. In younger patients with AML, HMA plus venetoclax yielded high response rates, measurable residual disease negativity, and a substantial proportion of patients proceeding to hematopoietic stem cell transplantation.

## INTRODUCTION

Acute myeloid leukemia (AML) is a clonal hematologic malignancy arising from the uncontrolled proliferation of genetically and epigenetically altered myeloid progenitors. Although AML predominantly affects older adults, with a median age at diagnosis of approximately 68 years,^1^ the disease spans all age groups, including pediatric, adolescent, and young adult populations, in whom distinct molecular and clinical characteristics have been described.^2^, ^3^ As a result, a clinically meaningful proportion of patients present at diagnosis as young and medically fit.

For decades, the treatment paradigm for younger patients with AML has relied on intensive induction chemotherapy, followed by consolidation therapy and in most intermediate‐ and adverse‐risk cases, allogeneic hematopoietic stem cell transplantation (HSCT).^4^, ^5^, ^6^, ^7^, ^8^, ^9^, ^10^, ^11^ Although this approach can achieve durable remissions, it is associated with substantial acute toxicity, prolonged cytopenias, infectious complications, and a significant impact on quality of life. Moreover, the biological heterogeneity of AML suggests that cytotoxic intensity alone may not be sufficient to optimize outcomes across molecular subgroups.

In patients considered unfit for intensive chemotherapy, the combination of hypomethylating agents (HMA) and venetoclax, a selective inhibitor of the antiapoptotic protein BCL‐2, has transformed the therapeutic landscape. This regimen promotes apoptosis in leukemic blasts by restoring mitochondrial priming and has demonstrated high rates of rapid and deep responses, including measurable residual disease (MRD) negativity.^1^ In the pivotal VIALE‐A trial, conducted in patients with a median age of 76 years (range, 49–91), azacitidine plus venetoclax significantly improved overall survival compared with azacitidine alone (median overall survival 14.7 vs. 9.6 months) and nearly doubled complete remission rates (36.7% vs. 17.9%).^12^ Subsequent analyses identified distinct molecular subsets, including *TP53*, *FLT3* internal tandem duplication (*FLT3‐ITD*), *RAS* pathway mutations, *NPM1*, and *IDH1/2*, highlighting the heterogeneity of response to HMA‐VEN and underscoring the importance of disease biology in shaping outcomes..^13^ These findings have been incorporated into the 2024 European LeukemiaNet (ELN) recommendations for patients receiving less‐intensive therapies.^14^

The favorable efficacy‐to‐toxicity profile of HMA plus venetoclax has prompted growing interest in its use beyond the elderly and unfit population, including younger patients as a potential alternative to conventional intensive chemotherapy. Early‐phase trials and real‐world studies suggest that this approach may induce high‐quality remissions while preserving performance status and facilitating transition to HSCT. The PARADIGM trial, an open‐label, multicenter, phase 2 randomized study, compared intensive chemotherapy (7+3 or CPX‐351) with azacitidine plus venetoclax in patients ≥18 years old; however, the exclusion of AML with core‐binding factor rearrangements, FLT3 mutations, or NPM1 mutations (unless ≥60 years old) limits the applicability of its results to the broader younger AML population.^15^

In light of the increasing use of HMA plus venetoclax in younger patients and the lack of definitive randomized data in this setting, we conducted a systematic review and meta‐analysis to synthesize the available evidence on clinical outcomes in patients <70 years old with AML treated with HMA and venetoclax, with a particular focus on response depth, survival, and the feasibility of bridging to allogeneic HSCT.

## MATERIALS AND METHODS

This systematic review and meta‐analysis was conducted in accordance with the Preferred Reporting Items for Systematic Reviews and Meta‐Analyses (PRISMA) guidelines,^16^ and the study protocol was registered in the International Prospective Register of Systematic Reviews (PROSPERO) registration. A comprehensive literature search was performed in February 2026 using Ovid MEDLINE (ALL: 2000 to present) and the Cochrane Library (Wiley), combining controlled vocabulary terms and free‐text keywords related to hypomethylating agents and venetoclax, including “azacitidine venetoclax,” “decitabine venetoclax,” “HMA venetoclax,” and “young AML.” To minimize publication bias, no restrictions were applied with respect to language or publication date, and abstracts presented at major hematology conferences, including the American Society of Hematology (ASH) Annual Meeting, were also screened. Studies were eligible for inclusion if they reported clinical outcomes in patients with acute myeloid leukemia treated with hypomethylating agents plus venetoclax and had a median patient age <70 years. The primary outcomes of interest were complete remission (CR/CRi) rate, 1‐year overall survival (OS), 1‐year event‐free survival (EFS), incidence of allogeneic HSCT, and rates of MRD negativity. Given the anticipated clinical and methodological heterogeneity across studies, pooled estimates were calculated using random‐effects models, with statistical heterogeneity assessed using Cochran’s *Q* test and quantified by the *I* ^2^ statistic. Comparisons between randomized controlled trials and real‐world evidence studies were descriptive and based on subgroup‐specific pooled estimates with corresponding 95% confidence intervals (CIs). All meta‐analyses and forest plots were generated using JASP software, version 0.95.4.^17^

## RESULTS

**FIGURE 1:**
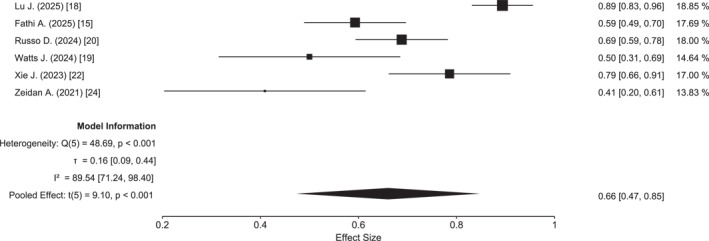
Forest plot of complete response rate.

**FIGURE 2:**
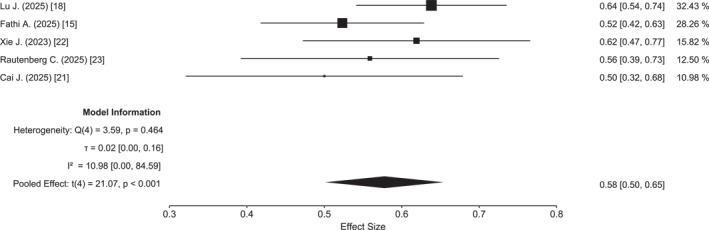
Forest plot of 1‐year event‐free survival.

**FIGURE 3:**
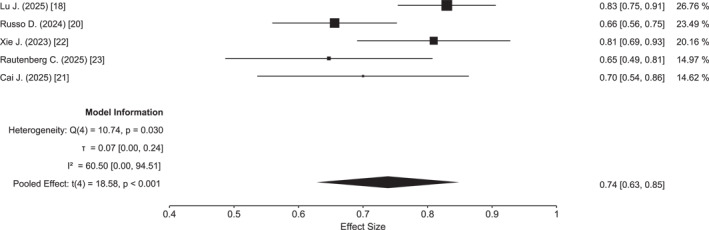
Forest plot of 1‐year overall survival.

**FIGURE 4:**
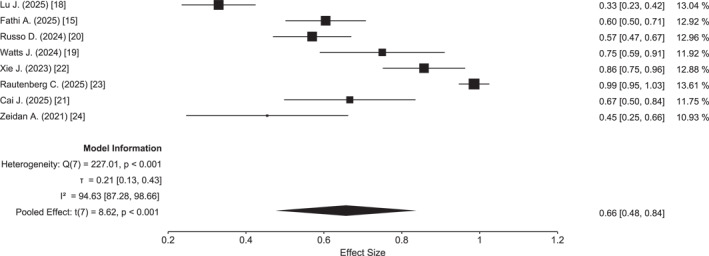
Forest plot of percentage of patients allotransplanted.

**FIGURE 5:**
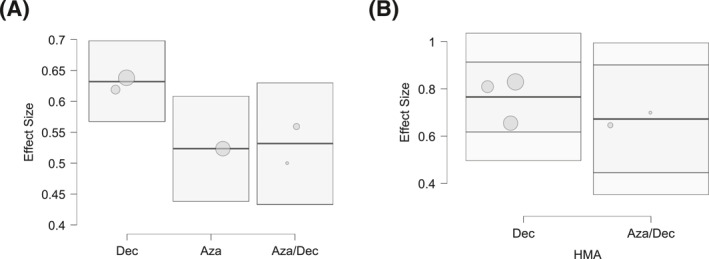
Bubble plots showing the effect of the drug in combination with venetoclax (azacitidine vs. decitabine vs. venetoclax/decitabine) on event‐free survival (left). On the right, the effect on overall survival of decitabine vs. venetoclax/decitabine.

A total of 56 records were retrieved from the Cochrane Library and 32 from PubMed; the study selection process is summarized in the PRISMA flow diagram (Figure S1). Overall, eight studies were included in the meta‐analysis, comprising two randomized controlled trials,^15^, ^18^ two prospective phase 2 studies,^19^, ^20^ and four real‐world evidence studies,^21^, ^22^, ^23^ including one registry‐based analysis using an electronic medical records database from the United States,^24^ for a total of 429 patients. The main characteristics of the included studies are summarized in Table 1, whereas the baseline clinical and molecular characteristics of patients are summarized in Table 2. Median patient age across studies ranged from 39 to 68 years, with a mean age of 54 years. The majority of patients were enrolled in studies conducted in China^18^, ^21^, ^22^ (166 patients), followed by the United States^15^, ^19^, ^24^ (136 patients) and Europe^20^, ^23^ (127 patients). With respect to the hypomethylating agent combined with venetoclax, three studies used decitabine,^18^, ^20^, ^22^ three used azacitidine,^15^, ^19^, ^24^ and two included mixed cohorts treated with either azacitidine or decitabine.^21^, ^23^ The pooled CR or CRi rate, available in six studies including 365 patients, was 66% (95% CI, 48%–85%), with substantial heterogeneity (*Q* = 49.9; *p* < .001; *I* ^2^ = 89%). The highest CR/CRi rate (89%) was reported in the study by Lu et al.,^18^ whereas the lowest (40.9%) was observed in the US database study^24^ (Figure 1). MRD negativity was evaluable in three studies; the study by Xie et al.^22^ was excluded from this analysis due to the limited sample size (*n* = 31). The pooled MRD‐negative rate was 66% (95% CI, 30%–100%), with considerable heterogeneity (*I* ^2^ = 85%) (Figure S2). The pooled 1‐year EFS, reported in five studies including 286 patients, was 58% (95% CI, 50%–65%), with minimal between‐study heterogeneity (*Q* = 3.9; *p* = .40; *I* ^2^ = 11%) (Figure 2). Similarly, the pooled 1‐year OS, available for five studies comprising 293 patients, was 74% (95% CI, 63%–85%), with low heterogeneity (*I* ^2^ = 18.6%) (Figure 3). The highest 1‐year OS (83%) was reported in the study by Lu et al.,^18^ whereas the lowest OS (65%) was observed in the US database study.^24^ All included studies reported the proportion of patients proceeding to HSCT, with a pooled transplantation rate of 66% (95% CI, 48%–84%). Data on relapse‐free survival stratified by transplantation status were inconsistently reported; where available, transplanted patients demonstrated prolonged remission duration, although direct pooled comparison was not feasible due to reporting heterogeneity. The lowest rate of HSCT (33%) was reported by Lu et al.,^18^ whereas the highest (99%) was observed in the study by Rautenberg et al.,^23^ reflecting the transplant‐focused nature of that cohort (Figure 4). Finally, meta‐regression analyses exploring the impact of patient age and hypomethylating agent backbone (azacitidine vs. decitabine) suggested a trend toward improved outcomes in studies employing decitabine, particularly for EFS and to a lesser extent for OS (Figure 5); additionally, studies enrolling patients with a median age ≤45 years demonstrated superior EFS compared with those including patients with a median age ≥47 years (Figure S3).

**TABLE 1:** Summary of the studies characteristics included into meta‐analysis.

Study	Authors and reference	Type	HMA	No. of patients	Median age, years	Median OS (months)	1‐year OS, %	1‐year EFS, %	30‐day mortality, %	60‐day mortality, %	Patients transplanted, %	CR/CRi, %	ORR (CR, CRi, CRh, MLFS), %	MRD negativity, %	TP53, %
China: Ven+Dec vs. IA	Lu 2025^18^	RCT	Dec	94	45		83.1	64.4	n/a	n/a	31 patients	89		80	
PARADIGM: AZA/Ven vs. IC	Fathi 2025^15^	RCT, phase 2	Aza	86	64	NR		53	0	0	61 (52 patients)	59	88	n/a	
Italy, GITMO	Russo 2024^20^	Phase 2	Dec	93	68		66		n/a	n/a	57	69		64	11
Miami, Florida	Watts 2024^19^	Phase 2	Aza	36	48	NR			6	n/a	68	59	63	53	11.1
China Phase 2 Dec/Ven	Xie 2023^22^	RWS	Dec	42	39	NR	82	61	0	n/a	86	79	81	76	
Cooperative Transplant Study Group Germany	Rautenberg 2025^23^	RWS	Aza/dec	34	64	28	65	57	n/a	n/a	100	58	67	65	35
ASXL1 Soochow University (China)	Cai 2025^21^	RWS	Aza/dec	30	47	25.3	70	50	n/a	n/a	66.7	76.7	72.8	66.7	n/a
EMR database in United States	Zeidan 2021^24^	EMR database	Aza	138	18–59	11.3			n/a	n/a	8	44.2			26/138

Abbreviations: aza, azacitidine; CR, complete response; CRh, Complete Rosponse with partial Hematologic recovery; CRi, CR with incomplete blood count recovery; dec, decitabine; EFS, event‐free survival; EMR, electronic medical records; HMA, hypomethylating agents; IA, Idarubicine and cytarabine; IC, intensive chemotherapy; MLFS, Morphologic Leukemia‐free state; MRD, measurable residual disease; n/a, not available; NR, not reported; ORR, overall response rate; OS, overall survival; PFS, progression‐free survival; RCT, randomized clinical trial; RWS, real‐world evidence study.

**TABLE 2:** Baseline clinical and molecular characteristics of patients included in the analyzed studies.

Study	Patients (*N*)	Mean age, years	ELN‐22 risk adverse (%)	Secondary AML (%)	ECOG ≥2 (%)	FLT3 (%)	NPM1 (%)	TP53 (%)	HSCT (%)
China: Ven+Dec vs. IA^18^	94	45	23	22	12	23	16	n/a	31 patients
PARADIGM: AZA/Ven vs. IC^15^	86	64	72			0	35^a^	11	61
Italy, GITMO^20^	93	68	53					11	57
Miami, Florida^19^	36	48	80.6	55	n/a	11	2.8	11.1	68
China Phase 2 Dec/Ven^22^	42	39				34		19	86
Cooperative Transplant Study Group Germany^23^	34	64	88	n/a	n/a	9	18	35	100
ASXL1 Soochow University (China)^21^	30	47	96.7	10	n/a	n/a	n/a	n/a	66.7
EMR database in United States^24^	138 (276 entire cohort)	18–59	Poor risk 71/276	105/276	42/276	6/276	34/276	26/138	8

*Note*: This table summarizes the main demographic, clinical, and molecular features reported across the studies included in the meta‐analysis of younger patients with acute myeloid leukemia treated with hypomethylating agents plus venetoclax. Variables include age distribution, ELN‐22 risk classification, performance status, frequency of key molecular alterations, and the proportion of secondary AML. Data are presented as reported in the original publications.

Abbreviations: AML, acute myeloid leukemia; aza, azacitidine; dec, decitabine; ECOG, Eastern Cooperative Oncology Group; EFS, event‐free survival; EMR, electronic medical records; HMA, hypomethylating agents; HSCT, hematopoietic stem cell transplantation; IC, intensive chemotherapy; n/a, not available.

^a^ *NPM1*/*IDH2*mut.

## DISCUSSION

There is growing interest in extending the therapeutic benefits of venetoclax, which is now the standard of care for elderly or unfit patients with AML,^12^ to younger and clinically fit populations. Initial efforts in this direction have largely focused on combining venetoclax with intensive chemotherapy (IC), particularly venetoclax plus FLAG‐Ida^25^, ^26^ or venetoclax combined with standard 7+3 daunorubicin and cytarabine regimens.^27^ An alternative and increasingly explored strategy is the use of HMA plus venetoclax as a less‐intensive, chemotherapy‐sparing approach in younger patients. To our knowledge, the present meta‐analysis represents the first systematic attempt to synthesize the available evidence on this treatment strategy specifically in younger patients with AML. Although biological fitness is widely regarded as a more clinically meaningful determinant of treatment intensity than chronological age, predefined age thresholds were applied in this meta‐analysis as pragmatic inclusion criteria, because of the inconsistent reporting of detailed fitness parameters and comorbidity indices across population‐based data sets. We recognize this as an inherent methodological constraint and underscore that, in routine clinical practice, therapeutic decisions should be guided primarily by physiological reserve and transplant eligibility rather than chronological age alone.^28^ The decision to administer HMA‐VEN in younger patients was not uniformly driven by chronological age alone but reflected heterogeneous clinical contexts, including comorbidity burden, physician discretion, and adverse‐risk disease biology. This variability limits definitive conclusions regarding optimal patient selection. For the purpose of this analysis, we applied an age cutoff of <70 years, resulting in a cohort with a median age of 54 years that is representative of younger, real‐world AML populations.

The most clinically relevant finding of this meta‐analysis is the favorable survival outcome observed with HMA plus venetoclax. The pooled 1‐year OS of 75% compares favorably with historical population‐based data, including the Surveillance, Epidemiology, and End Results database, which reported a 1‐year OS of approximately 62% among patients 40–59 years old treated between 2010 and 2017.^29^ In parallel, the pooled 1‐year EFS was approximately 60%, exceeding the EFS rates typically reported for younger patients treated with IC in large contemporary cohorts, which range from 40%–50% in patients under 60 years and decline further in older age groups.^4^, ^30^ Notably, EFS was the end point associated with the lowest heterogeneity in our analysis (*I* ^2^ = 3.2%), indicating a remarkable consistency of this outcome across studies despite differences in study design and patient populations. Although EFS has been debated as a surrogate end point in AML, it captures treatment failure, relapse, and death, and may therefore reflect the overall burden of therapy and health care resource utilization more comprehensively than OS alone.^31^

Beyond survival, HMA plus venetoclax demonstrated substantial antileukemic activity, with a pooled CR/CRi rate of 66% and a high rate of MRD negativity (69%). These findings suggest that deep remissions can be achieved even in younger patients without the use of intensive cytotoxic chemotherapy. The considerable heterogeneity observed for response and MRD outcomes likely reflects variability in response assessment methods, MRD detection techniques, timing of evaluations, and treatment schedules across studies. In this context, our meta‐regression analyses provide hypothesis‐generating insights, suggesting a trend toward improved EFS and, to a lesser extent, OS in studies using decitabine rather than azacitidine as the HMA backbone. Although prior studies have suggested that variations in venetoclax dosing or duration may have limited impact on efficacy,^32^, ^33^ the available data did not allow for a more granular analysis of these variables, underscoring the need for harmonized reporting in future trials.

Another key observation from this meta‐analysis is the high proportion of patients successfully bridged to HSCT, with a pooled transplantation rate of 66%. This finding supports the concept of HMA plus venetoclax as an effective induction strategy capable of achieving disease control sufficient to proceed to curative‐intent transplantation. Nevertheless, substantial variability was observed across studies, partly driven by differences in study design and intent. For example, in the Chinese randomized trial, only one‐third of patients ultimately underwent HSCT,^18^ suggesting a strategy aimed at achieving deep remissions and potentially deferring transplantation in patients attaining MRD negativity, thereby sparing them from transplant‐related toxicity and mortality. The Chinese randomized study by Lu J. et al.^18^ largely included younger patients with favorable‐risk and *CBF* AML, whereas PARADIGM predominantly enrolled intermediate‐ and adverse‐risk patients, reflecting regulatory constraints in the United States and the ethical necessity of incorporating approved *FLT3*‐inhibitors and gemtuzumab ozogamicin into standard induction regimens. However, in the absence of HSCT, HMA plus venetoclax generally requires continuous therapy until progression, and longer follow‐up is needed to determine whether durable treatment‐free remissions can be achieved in selected younger patients. Moreover, the trial by Lu et al.^18^ did not evaluate a fully nonintensive treatment paradigm; rather, it employed HMA‐VEN induction followed by protocol‐mandated intensive consolidation. Thus, observed survival and remission outcomes likely reflect a hybrid strategy, potentially attenuating differences between study arms.

Beyond efficacy, the tolerability and broader impact of HMA plus venetoclax merit consideration. Although robust pharmacoeconomic data comparing HMA/venetoclax with IC in younger patients are lacking, available evidence suggests that venetoclax‐based regimens may be cost‐effective in older, IC‐ineligible populations.^34^ Extrapolating these findings, a less‐intensive outpatient‐based approach may reduce hospitalization, mitigate treatment‐related morbidity, and alleviate the psychological and social burden associated with prolonged inpatient stays, including depression, anxiety, fatigue, and post‐traumatic stress symptoms.^35^, ^36^, ^37^ These aspects may be particularly relevant for younger, working‐age patients and deserve further prospective evaluation. Several limitations of this study should be acknowledged. The included evidence derives from heterogeneous sources, encompassing randomized trials, phase 2 studies, and real‐world analyses conducted across different geographic regions, with variability in patient selection, treatment protocols, and outcome definitions. Additionally, detailed molecular and genetic data were not consistently available, precluding patient‐level analyses that could refine risk stratification and treatment selection. Emerging data suggest that HMA plus venetoclax may reclassify some patients traditionally considered high risk by ELN criteria when treated with IC into more favorable prognostic categories when treated with less‐intensive regimens,^14^, ^38^ whereas other molecular subsets may derive greater benefit from IC‐based approaches. The PARADIGM trial exemplifies this complexity, having excluded patients with core‐binding factor rearrangements, FLT3 mutations, or NPM1 mutations, thereby highlighting the need for biomarker‐driven treatment allocation.^15^, ^39^ In conclusion, this meta‐analysis supports the feasibility and clinical activity of HMA plus venetoclax in younger patients with AML, demonstrating high response rates, frequent MRD negativity, encouraging survival outcomes, and a substantial capacity to bridge patients to HSCT. Although these findings do not support replacing intensive chemotherapy in favorable‐risk AML, where established IC‐based regimens remain highly effective, the data appear to become more supportive in adverse‐risk disease, where outcomes with standard IC are suboptimal and alternative strategies may be justified. Indeed, they provide a strong rationale for prospective randomized trials aimed to define the optimal role of HMA plus venetoclax within a risk‐adapted, biology‐driven treatment algorithm for younger patients with AML.

## AUTHOR CONTRIBUTIONS

**Salvatore Perrone**: Conceptsualization; investigation; writing—original draft; methodology; software; formal analysis. **Laura De Fazio**: Funding acquisition, visualization. **Sebastian Monachetti**: Project administration; writing—review and editing; validation; data curation; resources. **Matteo Molica**: Supervision; conceptualization; writing—review and editing; investigation; validation.

## CONFLICT OF INTEREST STATEMENT

The authors declare no conflicts of interest.

## Supporting information

Supplementary Material

## Data Availability

The data that support the findings of this study are available from the corresponding author on reasonable request.
