# Supplementary material for: Hypomethylating agents plus venetoclax in younger acute myeloid leukemia: Meta‐analysis of a shifting treatment paradigm
Source: Cancer. 2026 Mar 31;132(7):e70372. doi: 10.1002/cncr.70372 (PMC13036820; doi:10.1002/cncr.70372)
Supplement: Supplementary file 1 — Supplementary Material [file CNCR-132-e70372-s001.docx]

**Hypomethylating Agents Plus Venetoclax in Younger AML: Meta-Analysis of a Shifting Treatment Paradigm**

Salvatore Perrone^1,^ Laura De Fazio ^2^, Sebastian Monachetti^3*^, Matteo Molica ^2^

1. Department of Hematology, S. M. Goretti Hospital, Polo Universitario Pontino, Latina, Italy.

2. Department of Hematology-Oncology, Azienda Universitaria Ospedaliera Renato Dulbecco, 88100 Catanzaro, Italy.

3. Department of Translational and Precision Medicine, Sapienza University, Rome, Italy.

* Correspondence to: Sebastian Monachetti [sebastian.monachetti@uniroma1.it](mailto:sebastian.monachetti@uniroma1.it)

Department of Translational and Precision Medicine, Sapienza University, Rome, Italy. 00173

Via Benevento,6

**SUPPLEMENTAL MATERIAL**

**Figure 1 Supplemental** Prisma flow chart of the screening process

**
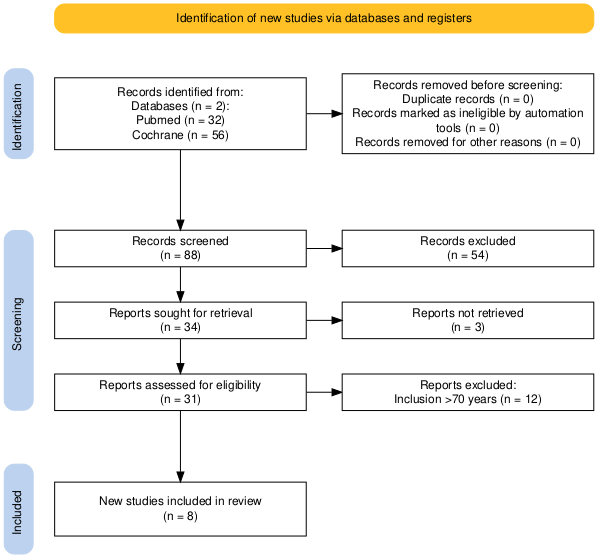
**

Haddaway, N. R., Page, M. J., Pritchard, C. C., & McGuinness, L. A. (2022). PRISMA2020: An R package and Shiny app for producing PRISMA 2020-compliant flow diagrams, with interactivity for optimised digital transparency and Open Synthesis Campbell Systematic Reviews, 18, e1230. https://doi.org/10.1002/cl2.1230

**Figure 2 Supplemental** Forest plot of MRD negativity.


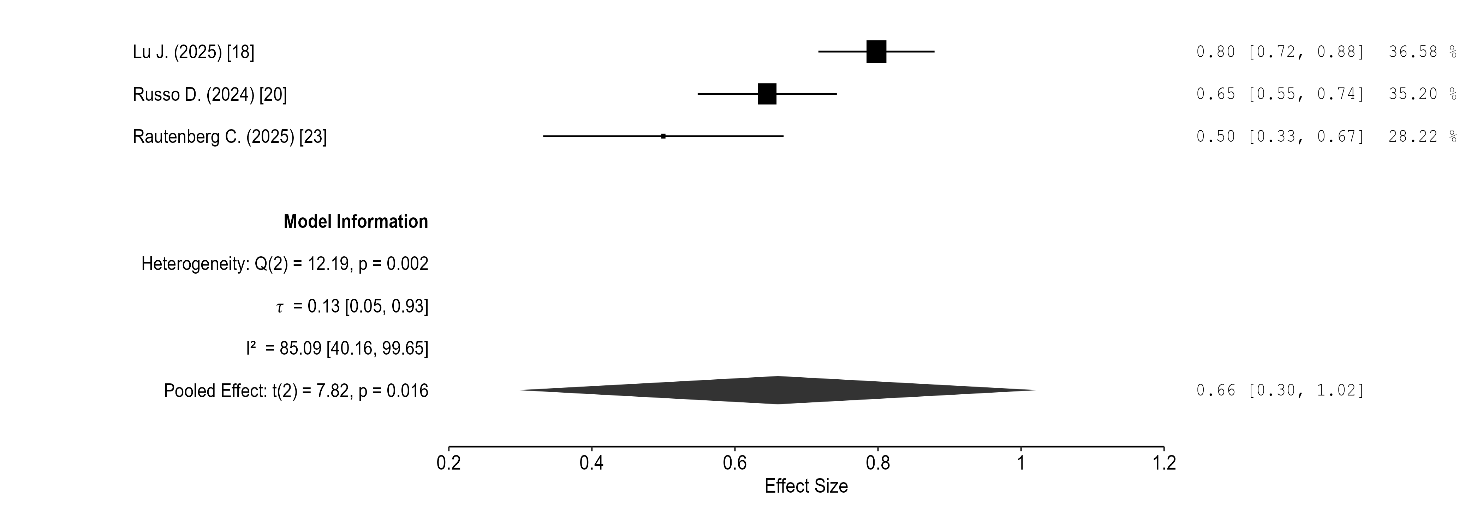


**Figure 3 Supplemental** Bubble plots showing the effect of the median age on Event-free Survival EFS.

**
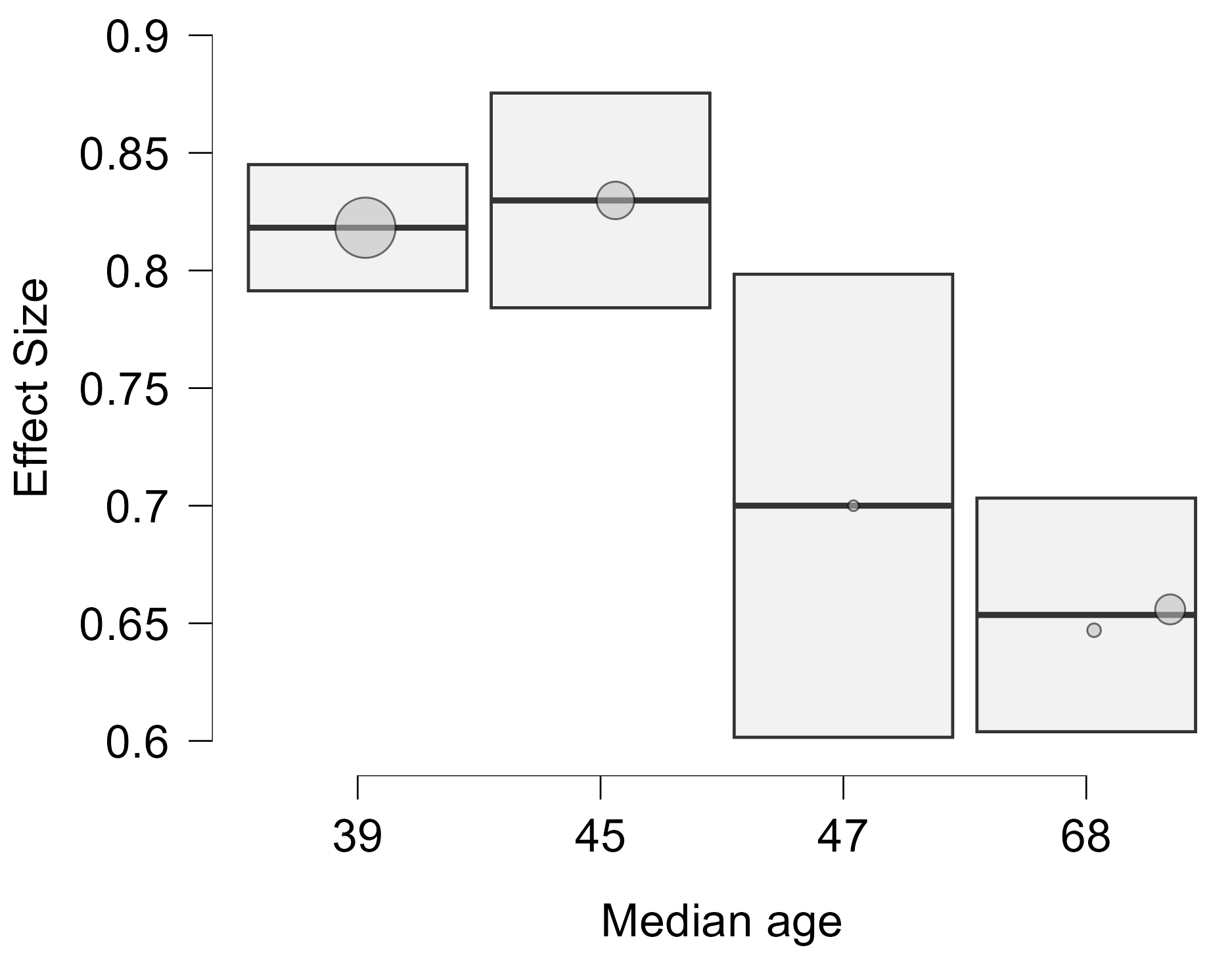
**
